# Adult mesenchymal stem cell ageing interplays with depressed mitochondrial Ndufs6

**DOI:** 10.1038/s41419-020-03289-w

**Published:** 2020-12-15

**Authors:** Yuelin Zhang, Liyan Guo, Shuo Han, Ling Chen, Cheng Li, Zhao Zhang, Yimei Hong, Xiaoxian Zhang, Xiaoya Zhou, Dan Jiang, Xiaoting Liang, Jianxiang Qiu, Jinqiu Zhang, Xin Li, Shilong Zhong, Can Liao, Bin Yan, Hung-Fat Tse, Qizhou Lian

**Affiliations:** 1 Department of Emergency Medicine, Guangdong Provincial People’s Hospital, Guangdong Academy of Medical Sciences, Guangzhou, Guangdong China; 2 Guangzhou Women and Children’s Medical Centre, Guangzhou Medical University, Guangzhou, Guangdong China; 3 Department of Medicine, Li Ka Shing Faculty of Medicine, the University of Hong Kong, Hong Kong SAR, China; 4 Clinical Translational Medical Research Center, Shanghai East Hospital, Tongji University School of Medicine, Shanghai, China; 5 Guangdong Cardiovascular Institute, Guangdong Provincial People’s Hospital, Guangdong Academy of Medical Sciences, Guangzhou, Guangdong China; 6 Department of Computer Science, Faculty of Engineering, the University of Hong Kong, Hong Kong SAR, China; 7 The State Key Laboratory of Pharmaceutical Biotechnology, the University of Hong Kong, Hong Kong SAR, China

**Keywords:** Ageing, Stem-cell research

## Abstract

Mesenchymal stem cell (MSC)-based therapy has emerged as a novel strategy to treat many degenerative diseases. Accumulating evidence shows that the function of MSCs declines with age, thus limiting their regenerative capacity. Nonetheless, the underlying mechanisms that control MSC ageing are not well understood. We show that compared with bone marrow-MSCs (BM-MSCs) isolated from young and aged samples, NADH dehydrogenase (ubiquinone) iron-sulfur protein 6 (Ndufs6) is depressed in aged MSCs. Similar to that of Ndufs6 knockout (Ndufs6^−/−^) mice, MSCs exhibited a reduced self-renewal and differentiation capacity with a tendency to senescence in the presence of an increased p53/p21 level. Downregulation of Ndufs6 by siRNA also accelerated progression of wild-type BM-MSCs to an aged state. In contrast, replenishment of Ndufs6 in Ndufs6^−/−^-BM-MSCs significantly rejuvenated senescent cells and restored their proliferative ability. Compared with BM-MSCs, Ndufs6^−/−^-BM-MSCs displayed increased intracellular and mitochondrial reactive oxygen species (ROS), and decreased mitochondrial membrane potential. Treatment of Ndufs6^−/−^-BM-MSCs with mitochondrial ROS inhibitor Mito-TEMPO notably reversed the cellular senescence and reduced the increased p53/p21 level. We provide direct evidence that impairment of mitochondrial Ndufs6 is a putative accelerator of adult stem cell ageing that is associated with excessive ROS accumulation and upregulation of p53/p21. It also indicates that manipulation of mitochondrial function is critical and can effectively protect adult stem cells against senescence.

## Introduction

Preclinical and clinical trials have revealed mesenchymal stem cell (MSC)-based therapy to be a promising therapeutic strategy for many diseases^1–5^. Compared with other types of stem cells currently under investigation, MSCs have several appreciable advantages, including easy isolation and being highly expandable with multilineage differentiation potential and low immunogenicity. Nonetheless an increasing body of evidence has demonstrated that their benefits decline during the aging process. Prolonged ex vivo cell culture of MSCs isolated from ageing donors show a senescent state, and reduced proliferation and impaired differentiation capacity, thus limiting their clinical application^6,7^ although the molecular network governing this senescence largely remains elusive. Exploration of the mechanisms that underlie cellular senescence is urgently needed.

To date, several potential mechanisms, including telomere shortening^8^, impaired autophagy^9^, and increased reactive oxygen species (ROS) have been reported to mediate the senescence of MSCs. Importantly, in addition to these factors stem cell senescence is strongly associated with mitochondrial dysfunction. One theory is that dysfunctional mitochondria accumulate with age^10^, while another proposes that mitochondrial dysfunction can directly induce stem cell ageing and impair autophagic function with a consequent decline in their regenerative function^11,12^. Mitochondria can no longer be viewed as simple bioenergy factories, but rather as platforms for intracellular signaling, regulators of innate immunity, and modulators of stem cell activity. In turn, each of these properties provides clues as to how mitochondria might regulate aging and age-related diseases^13^. Compared with young MSCs, senescent MSCs exhibit increased ROS, largely as a result of mitochondrial structural remodeling^14^. Inflammation-induced ROS leads to MSC senescence by upregulating the expression of miR-155 that in turn suppresses the expression of redox genes including Nfe2l2, Sod1, and Hmox1^15^. Moreover, cholesterol reduces senescence in bone marrow-MSCs (BM-MSCs) by inhibiting the ROS/p53/p21^Cip1/Waf1^ pathway, indicating that ROS plays a very important role in the senescence of MSCs^16^. To the best of our knowledge, mitochondria are the major source of ROS and structural alterations to mitochondria result in their excessive level^17^.

Mitochondrial dysfunction with consequent increased ROS level is closely related to cellular senescence^18,19^. Among the mitochondrial complexes in the electron transport chains, mitochondrial complex I is most commonly impaired in aged MSCs^20,21^. It is the largest component of the mitochondrial respiratory chain and the major entry point for electrons into the process of oxidative phosphorylation (OXPHOS). The OXPHOS machinery in mitochondria has five complexes, including complex I, complex II (succinate dehydrogenase), complex III (cytochrome bc1 complex), complex IV (cytochrome c oxidase, COX), and complex V (ATP synthase)^22^. These complexes are localized within the inner mitochondrial membrane. Complex I mediates ROS generation, and complex I defect leads to increased ROS production and decreased antioxidant defenses, thus impairing mitochondrial function^23–25^. Although complex I consists of at least 45 subunits, little is known about which subunit contributes to its stability and activity in MSC ageing.

NADH dehydrogenase (ubiquinone) iron-sulfur protein 6 (Ndufs6) is one of the major accessory subunits of complex I. Loss of this subunit caused by mutations has been reported to lead to fetal disease via mitochondrial complex I deficiency^26^. A previous study also demonstrated that Ndufs6 knockdown resulted in complex I instability and functional deficiency, leading to renal impairment due to increased ROS generation^23^. Given that Ndufs6 has been linked to complex I function, we hypothesized that Ndufs6 plays putative roles in MSC ageing and that Ndufs6 mediates cell senescence by regulating complex I function. Whether and how Ndufs6 mediates MSC senescence has nevertheless not been determined. In this study, we reveal that Ndufs6 plays a critical role in the regulation of BM-MSC senescence, and downregulation of Ndufs6 accelerates BM-MSC senescence via complex I deficiency with consequent increased ROS generation and activation of the p53/p21 signaling pathway.

## Materials and methods

### mRNA expression analysis

Five public mRNA expression microarray datasets were utilized to identify mRNA expression changes to mRNA of Ndufs1–8 genes during the aging process in humans and mice. They included (1) GSE9593 (GEO accession number) representing early and senescent passages of human BM-derived MSCs. (2) GSE35959 representing human MSC aging and primary osteoporosis. (3) GSE70376 comparing muscle stem cells (satellite cells) in homeostatic conditions and after cardiotoxin injury. (4) GSE47177 comparing quiescent satellite cells from mouse hindlimb muscle mouse uninjured young and old ones. (5) GSE56560 comparing young and senescent human MSCs from two donors. The processed data of these studies were extracted from GEO database. Expression data of Ndufs1–8 genes were selected for the linear regression analysis.

### Characterization of BM-MSCs and Ndufs6^−/−^-BM-MSCs

All animal experiments were approved by the Committee on the Use of Live Animals in Teaching and Research of the University of Hong Kong (CULATR 203871–16). Gene-Trap (Ndufs6^gt/gt^) mice were kindly provided by Dr. David R. Thorburn’s Lab (Murdoch Children’s Research Institute, Australia)^27^. The gene-trap embryonic stem cell lines (AR01380) were derived from129/Ola strain. The chimeric mice were bred to C57BL/6 background. All mice were produced in the mixed genetic background of C57BL/6 and 129/Ola. To acquire Ndufs6^−/−^ (KO) and Ndufs6^+/+^ wild-type (WT) offspring, heterozygous parents were mated, and genotyping were characterized^27^. BM-MSCs and Ndufs6^−/−^-BM-MSCs were isolated from WT and Ndufs6^−/−^-mice (6–8 weeks old), as previously described^28^. Briefly, the femurs and tibias of WT and KO mice were harvested, and then flushed with PBS using a 23-gauge syringe. After filtering with a 70-mm filter and BM cells were plated in culture dishes with complete DMEM medium containing DMEM/low glucose (Gibco), 10% FBS (Life Technologies, 16000), 5 ng/mL epidermal growth factor (PeProTech, AF-100–15), 5 ng/mL fibroblast growth factor (PeProTech, 100–18B), 0.1 mM 2-mercaptoethanol (Life Technologies, 21985023), Glutamax (Life Technologies, 35050061), NEAA (Life Technologies, 11140050), and penicillin–streptomycin (Life Technologies, 15140122) and then incubated at 37 °C with 5% CO_2_ in a humidified chamber. Three hours later, non-adherent cells were removed and the adherent cells were cultured in complete DMEM medium. Surface marker profiling and differentiation capacity of MSCs were examined, as previously reported^29^. Briefly, 1.5 × 10^5^ MSCs were stained with the following antibodies: Sca-1 (eBioscience, 11–5981–82), CD34 (eBioscience, 11–0341–82), CD45 (eBioscience, 48–0451–82), CD90 (eBioscience, 11–0903–82), and CD105 (eBioscience, PA5–12511). Isotype-matched antibodies were used as negative control. Finally, data were analyzed by collecting 30,000 events on Beckman Coulter FC500 using CXP Analysis 2.0 software. The differentiation capacity of adipogenesis, chondrogenesis, and osteogenesis of BM-MSCs and Ndufs6^−/−^-BM-MSCs at passages 3 and 4 was evaluated using the appropriate commercial kit (adipogenesis: Life Technologies, A1007001; chondrogenesis: Life Technologies, A1007101; osteogenesis: Life Technologies, A1007201) according to the protocol. The differentiation medium was changed every 3 days.

### RT-PCR

Genes related to adipogenesis (LPL and Pparg), chondrogensis (Acan and Col2a1), and osteogenesis (Bglap and Alpl) were detected by real-time PCR (RT-PCR), as previously reported^29^. Total RNA was extracted using RNeasy kit (Qiagen, 74104). A total of 1 µg mRNA was reverse-transcribed using PrimeScript RT reagent kit (Takara, RR047A). The program for PCR was as follows: 95 °C for 10 min, 40 cycles of 95 °C for 15 s, and 60 °C for 1 min. The primers for mRNA were as follows: LPL (F: TAACTGCCACTTCAACCACA, R: AATCAGCGTCATCAGGAGA), Acan (F: CAAGAAATCGAATCCCCAAATC, R: ACTTAGTCCACCCCTCCTCACAT), Pparg (TTTCAAGGGTGCCAGTTTCG, R: GGTGGGACTTTCCTGCTAATACAA), Col2a1 (F: CCGAGTGGAAGAGCGGAGAC, R: CAGTGGACAGTAGACGGAGGAAAG), Bglap (F: CCCAGACCTAGCAGACACCATG, R: TGTTCACTACCTTATTGCCCTCC), Alpl (F: TGGACGGTGAACGGGAAAA, R: AGCACAGCCAGTGGAAGCAG), Ndufs6 (F: GTACGACGCGTGGGGTC, R: CGACACTTGAACCCCGAAAC), p53 (F: CCCCTGTCATCTTTTGTCCCT, R: AGCTGGCAGAATAGCTTATTGAG), p21 (F: CCTGGTGATGTCCGACCTG, R: CCATGAGCGCATCGCAATC), and β-actin (F: AGAGGGAAATCGTGCGTGAC, R: TGCTGGAAGGTGGACAGTGAG). The fold change for mRNA relative to β-actin was determined by the formula: 2^−ΔΔCt^.

### SA-β-gal staining

SA-β-gal staining was performed according to the manufacturer’s instructions (Cell Signaling Technology). Briefly, after washing with PBS, MSCs were fixed with fixative solution for 15 min at room temperature and then stained with SA-β-gal staining solution at 37 °C overnight. Senescent MSCs were stained blue and were photographed. The percentage was calculated from five different view fields of each sample in three independent experiments.

### Transmission electron microscope

The mitochondrial morphology and autophagosomes of BM-MSCs and Ndufs6^−/−^-BM-MSCs were evaluated by a transmission electron microscope (TEM), as previously described^30^. The samples were photographed randomly under a TEM (Hitachi, H-7650). Finally, the mitochondrial length and number of autophagosomes were calculated for five different view fields of each group in three independent experiments.

### Immunofluorescence staining

Different groups of MSCs were cultured in 24-well plates with glass coverslips and fixed with 4% PFA, permeabilized with 0.1% Triton‐100, and then blocked by 5% bovine serum albumin. Subsequently, cells were incubated with rabbit anti-Ki-67 (Abcam, ab15580) at 4 °C overnight followed by fluorescent secondary antibody. Finally, the sample was mounted with DAPI, and ten random fields photographed. The percentage of Ki-67-positive cells was calculated.

### siRNA intervention

To knockdown of Ndufs6 in MSCs, Ndufs6-siRNA (Santa Cruz, sc-149888) and control siRNA (Santa Cruz, sc-37007) were used to transfect MSCs with a Lipofectamine RNAiMAX Reagent Kit (Invitrogen, 13778–075) at a standardized MOI (multiplicity of infection) of 5 according to the protocol. After 72 h, MSCs were harvested and the silencing efficiency was evaluated by western blotting.

### Western blotting

The proteins of the different groups of MSCs were extracted and the concentration measured by Bradford. A total amount of 25 μg protein of each sample was loaded, separated by SDS/PAGE, and then transferred to a PVDF membrane. The membrane was blocked with 5% fat-free milk in TBST and then incubated with the following antibodies: OXPHOS (Abcam, ab110413), Ndufs6 (GeneTex, GTX88043), p53 (Santa Cruz, SC-98), p21 (Santa Cruz, SC-271532), and GAPDH (Santa Cruz, SC-137179) at 4 °C overnight. After washing with TBST, the membrane was incubated with horseradish peroxidase-conjugated secondary antibodies (1:10,000; Santa Cruz) at room temperature for 1 h and then allowed to develop.

### Measurement of ROS

Intracellular and mitochondrial ROS were measured by 2′,7′-dichlorodihydrofluorescein diacetate (DCFH-DA) and Mito-Sox staining, respectively. Briefly, MSCs from different experimental groups were cultured in 24-well plates with glass coverslips. Then MSCs were incubated with 10 μM DCFH-DA (Invitrogen, C369) or 5 μM Mito-Sox (Invitrogen, M36008) for 10 min at 37 °C in the dark. Finally, the sample was photographed randomly and fluorescence intensity calculated for five different view fields of each group in three independent experiments, using Image J software. Moreover, the ROS level was also examined by flow cytometry. Briefly, MSCs were grown to 80% confluence in 6-cm culture dish, and incubated in 10 μM DCFH-DA or 5 μM Mito-Sox with fluorescence-activated cell-sorting staining buffer at 37 °C for 10 min. After incubation, MSCs were washed twice with PBS and analyzed by flow cytometry (FC500; Beckman Coulter). A shift to the right indicates increased ROS levels. BM-MSCs were treated with 10 mM H_2_O_2_ for 6 h for DCFH-DA staining or 100 mM antimycin A for 30 min for Mito-SOX staining as positive control.

### TMRM staining

Mitochondrial membrane potential (MMP) was detected by tetramethylrhodamine ethyl ester perchlorate (TMRM, Invitrogen, T668) according to the protocol. Briefly, MSCs from different experimental groups were cultured in 24-well plates with glass coverslips and then stained with 50 nM TMRM for 10 min. Finally, the sample was photographed randomly and fluorescence intensity calculated for five view fields of each group in three independent experiments, using Image J software. Moreover, we also analyzed the MMP with TMRM staining, using flow cytometry. BM-MSCs were treated with 10 μM carbonyl cyanide-*p*-trifluoromethoxyphenylhydrazone (FCCP) for 1.5 h as positive control.

### AAV transfection

The adeno-associated virus-Ndufs6 (AAV2-Ndufs6) plasmid was constructed as previously reported^31^. Briefly, the open reading frame of human Ndufs6 (NM_004553.4) was amplified and cloned into an AAV2 core vector (Fig. S1). After the Sanger sequencing, the AAV2-Ndufs plasmids were packaged for viral products (BioWit Technologies, China). Ndufs6^−/−^-BM-MSCs were infected using AAV2-Ndufs6 virus with an MOI of 20,000. The empty AAV2-infected Ndufs6^−/−^-BM-MSCs cells were used as negative control. The infection efficiency was confirmed by western blotting for Ndufs6 at 72 h post infection.

### Oxygen consumption rate measurement

The mitochondrial function of MSCs was evaluated by seahorse XFp analyzer as previously described (Agilent Technologies, RRID: SCR_013575)^1^. Briefly, four bioenergetics measurements were detected: (1) basal reparation—in assay medium containing 4.5 g/L glucose; (2) inhibition of ATP synthase—using oligomycin (1 μM), ATP synthesis turnover, and respiration driving proton leak; (3) maximal mitochondrial respiratory capacity—post treatment with FCCP (2 μM); and (4) non-mitochondrial respiration—following treatment with complex I inhibitor, rotenone (2 μM).

### Statistical analysis

All values are expressed as mean ± SD. Statistical tests were performed using Prism version 5.0c (GraphPad Software). Statistical significance was determined by independent-samples *T* test between two groups or analysis of variance followed by Bonferroni test between more than two groups. A *p* < 0.05 was considered significant.

## Results

### Accessory subunit of mitochondrial complex I Ndufs6 is downregulated in aged MSCs

**Fig. 1:**
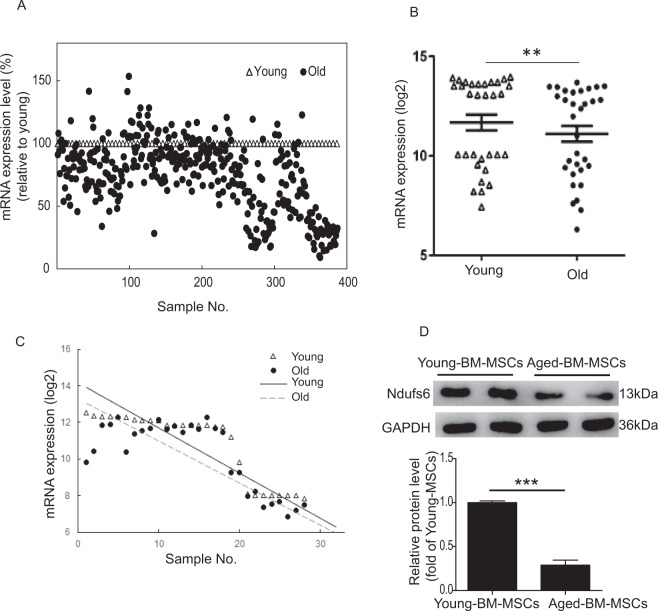
mRNA expression change of Ndufs6 during aging in human or mouse MSCs. mRNA expression data were extracted from five microarray datasets representing young vs. old/senescent cells and tissues or early stages of MSC differentiation. **A** Scatter plot for mRNA expression (relative to young) for all subunits (Ndufs1–8) of mitochondria complex assembly I. **B** mRNA expression difference of Ndufs6 between young and old cells or tissues (*n* = 32). **C** Linear regression of mRNA expression of Ndufs6 between young and old cells or tissues (*n* = 30). **D** The protein level of Ndufs6 in young-BM-MSCs and aged-BM-MSCs isolated from mice was determined by western blotting. Significant difference between young and old cells or tissues, **p < 0.01  ***p < 0.001.

To examine the relationship between accessory subunits of mitochondrial complex I and aging, we first extracted gene expression of Ndufs1–8 from five publicly available microarray datasets representing young versus old/senescent cells and tissues. The datasets included (1) GSE9593 (GEO accession number) representing early and senescent passages of human BM-derived MSCs. (2) GSE35959 representing human MSCs from young, aging, and primary osteoporosis. (3) GSE70376 representing muscle stem cells (satellite cells) in homeostatic conditions and regenerative cardiotoxin injury-stimulated muscle stem cells from aged mice. (4) GSE47177 compared quiescent muscle stem cells (satellite cells) from uninjured mice, young and old. (5) GSE56560 representing passage 9 human MSCs from two donors at days 2 and 7 of culture. In all five datasets, we observed a similar decrease in mRNA expression of all eight Ndufs subunits during the aging process (Fig. S2). In particular, expression of Ndufs3, 4, and 6 was consistently and prominently decreased. We further analyzed the difference between Ndufs6 mRNA from young and old/senescent cells and tissues using linear regression. A scatter plot for mRNA expression (relative to young) of all Ndufs subunits of mitochondria complex assembly I is shown in Fig. 1A. The mRNA expression of Ndufs6 is significant different between young and old cells or tissues (Fig. 1B). Linear regression of mRNA expression in the young is larger than that of the old samples (Fig. 1C). RT-PCR results showed that the mRNA level of Ndufs6 was much lower in aged-BM-MSCs derived from mice than young-BM-MSCs (Fig. S3). Further, the protein level of Ndufs6 was greatly reduced in aged-BM-MSCs derived from mice compared with young-BM-MSCs (Fig. 1D). This preliminary finding indicates that a dominant decline in expression of Ndufs6 may be a pivotal factor that provokes MSC ageing or a consequence in aged MSCs.

### Reduced differentiation potential of BM-MSCs in the absence of Ndufs6

Given the important role of Ndufs6 in complex I activity and MSC ageing, we next characterized BM-MSCs from WT and Ndufs6^−/−^ mice^27^. First, we examined the cell death between BM-MSCs and Ndufs6^−/−^-BM-MSCs. Trypan blue staining showed that there was no difference of cell death between BM-MSCs and Ndufs6^−/−^-BM-MSCs (data not shown). Next, surface markers of BM-MSCs and Ndufs6^−/−^-BM-MSCs were examined by fluorescence cytometry. The results showed that BM-MSCs and Ndufs6^−/−^-BM-MSCs had similar surface markers, i.e., Sca (+), CD90 (+), CD105 (+), CD45 (−), and CD34 (−) (Fig. S4A). Western blotting showed that Ndufs6 was highly expressed in BM-MSCs, but not in Ndufs6^−/−^BM-MSCs, confirming the absence of Ndufs6 in Ndufs6^−/−^-BM-MSCs (Fig. S4B). The differentiation capacity of BM-MSCs and Ndufs6^−/−^-BM-MSCs for adipogenesis, chondrogenesis, and osteogenesis was evaluated by staining with oil red, alcian blue, and alizarin red, respectively. Compared with BM-MSCs, adipogenic, chondrogenic, and osteogenic differentiation capacity of Ndufs6^−/−^-BM-MSCs was significantly reduced (Fig. S4C). To further verify their differentiation capacity, the genes related to adipogenesis (LPL and Pparg), chondrogenesis (Acan and Col2a1), and osteogenesis (Bglap and Alpl) were detected by RT-PCR. The mRNA expression of LPL, Pparg, Acan, Col2a1, Bglap, and Alpl was greatly decreased in Ndufs6^−/−^-BM-MSCs in differentiation conditions compared with that of BM-MSCs (Fig. S4D–F). These results indicate that the differentiation functions of Ndufs6^−/−^-BM-MSCs dramatically declined compared with WT BM-MSCs.

### Ndufs6 low-BM-MSCs exhibits increased cellular senescence

**Fig. 2:**
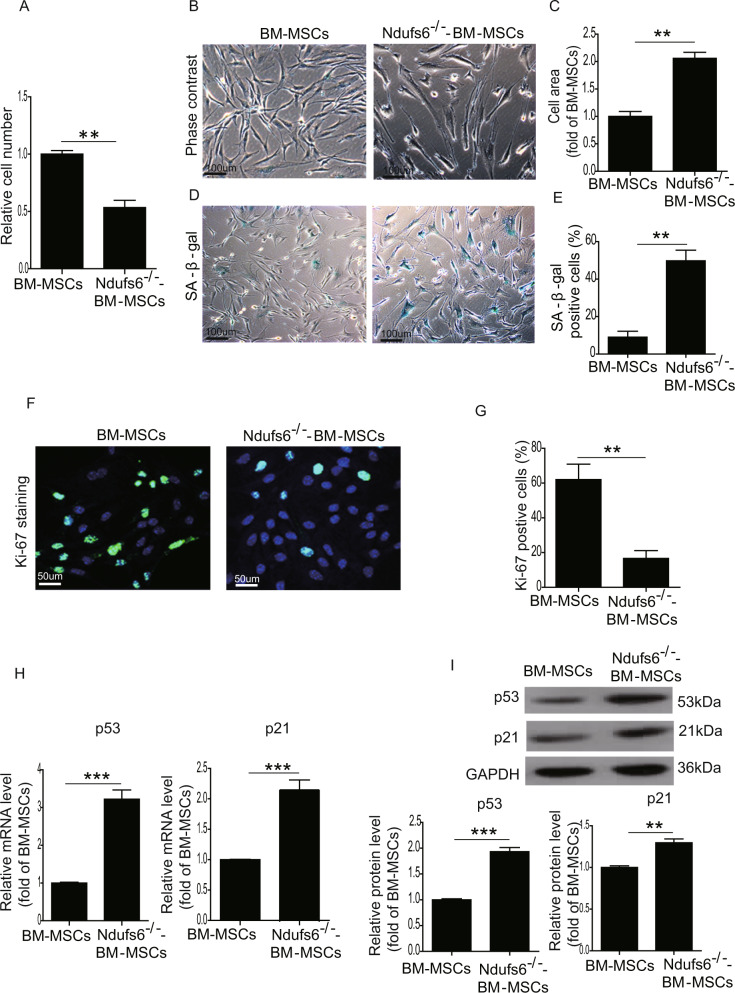
Ndufs6^−/−^-BM-MSCs exhibit increased cellular senescence. **A** The same number of BM-MSCs and Ndufs6^−/−^-BM-MSCs was cultured, and cells counted and presented 2 days later. **B** The typical cellular morphology of BM-MSCs and Ndufs6^−/−^-BM-MSCs was observed. **C** The cell area of BM-MSCs and Ndufs6^−/−^-BM-MSCs was quantified. **D** SA-β-gal staining showing β-gal-positive cells in BM-MSCs and Ndufs6^−/−^-BM-MSCs. **E** The β-gal positivity in BM-MSCs and Ndufs6^−/−^-BM-MSCs was quantified by calculating the ratio of stained cells to total cells. **F** The proliferation of BM-MSCs and Ndufs6^−/−^-BM-MSCs was assessed by Ki-67 staining. **G** The Ki-67 positivity in BM-MSCs and Ndufs6^−/−^-BM-MSCs was quantified by calculating the ratio of stained cells to total cells. **H** The mRNA level of p53 and p21 in BM-MSCs and Ndufs6^−/−^-BM-MSCs was determined by RT-PCR. **I** The protein level of p53 and p21 in BM-MSCs and Ndufs6^−/−^-BM-MSCs was determined by western blotting. Results are presented as mean ± SD. *n* = 3. **p < 0.01; ***p < 0.001.

Increasing evidence has shown that dysfunction of MSCs is caused by premature ageing of stem cells. We therefore compared the cellular senescence of BM-MSCs and Ndufs6^−/−^-BM-MSCs. We first examined cellular proliferation by counting cell numbers of BM-MSCs and Ndufs6^−/−^-BM-MSCs, and confirmed that the cellular proliferation of Ndufs6^−/−^-BM-MSCs was significantly reduced (Fig. 2A). BM-MSCs exhibited a healthy spindle shape, whereas Ndufs6^−/−^-BM-MSCs showed an enlarged and fried egg-like shape (Fig. 2B) with a notably increased cell area compared with BM-MSCs (Fig. 2C). Moreover, SA-β-gal staining showed that the number of SA-β-gal-positive cells was greatly enhanced in Ndufs6^−/−^-BM-MSCs compared with BM-MSCs (Fig. 2D, E). To further verify the senescence of Ndufs6^−/−^-BM-MSCs, we performed Ki-67 staining to examine their proliferative capacity and showed that the of number Ki-67-positive cells was much lower in Ndufs6^−/−^-BM-MSCs than BM-MSCs (Fig. 2F, G). RT-PCR results showed that the mRNA level of p53 and p21, senescence-associated markers, was significantly elevated in Ndufs6^−/−^-BM-MSCs compared with BM-MSCs (Fig. 2H). Moreover, western blotting analysis also showed that the protein level of p53 and p21 was much higher than BM-MSCs (Fig. 2I). These results suggest that Ndufs6 may play a critical role in regulating cellular senescence.

### Knockdown of Ndufs6 accelerates cellular senescence in BM-MSCs

**Fig. 3:**
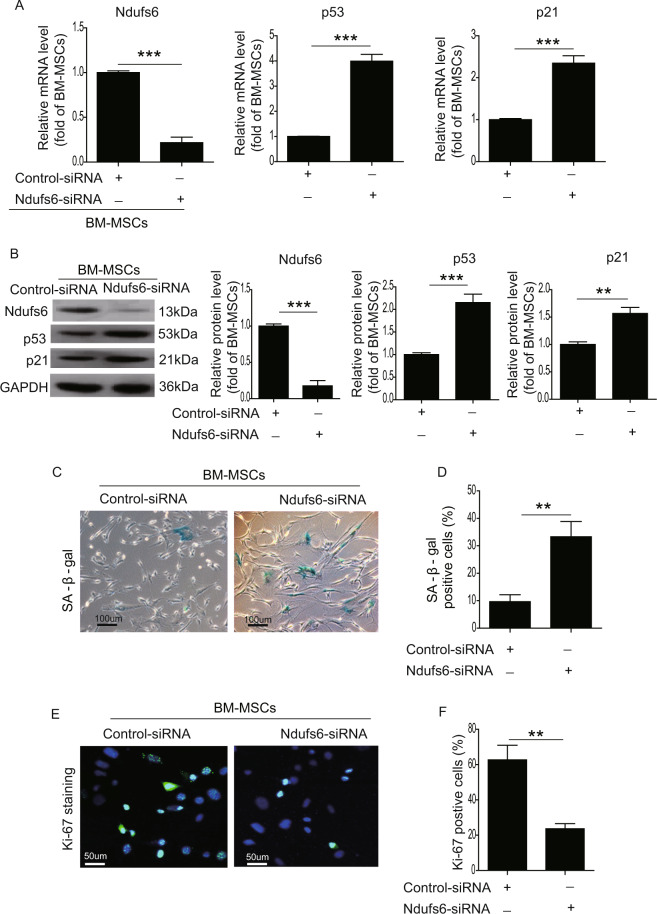
Ndufs6 knockout accelerates cellular senescence in BM-MSCs. **A** The mRNA level of Ndufs6, p53, and p21 was detected in BM-MSCs and Ndufs6-siRNA-treated BM-MSCs. **B** The protein level of Ndufs6, p53, and p21 was detected in BM-MSCs and Ndufs6-siRNA-treated BM-MSCs. **C** SA-β-gal staining showing β-gal-positive cells in BM-MSCs and Ndufs6-siRNA-treated BM-MSCs. **D** The β-gal positivity in BM-MSCs and Ndufs6-siRNA-treated BM-MSCs was quantified by calculating the ratio of stained cells to total cells. **E** The proliferation of BM-MSCs and Ndufs6-siRNA-treated BM-MSCs was assessed by Ki-67 staining. **F** The Ki-67 positivity in BM-MSCs and Ndufs6-siRNA-treated BM-MSCs was quantified by calculating the ratio of stained cells to total cells. Results are presented as mean ± SD. *n* = 3. **p < 0.01; ***p < 0.001.

To further explore the relationship between Ndufs6 and cellular senescence, we performed experiments with loss- and gain-of-function of Ndufs6 in MSCs. First, we used Ndufs6-siRNA to treat BM-MSCs. RT-PCR results showed that the mRNA level of Ndufs6 was markedly reduced, whereas the mRNA level of p53 and p21 was significantly enhanced in Ndufs6-siRNA-treated BM-MSCs compared with BM-MSCs (Fig. 3A). Similarly, western blotting also showed that the protein level of Ndufs6 was markedly decreased in Ndufs6-siRNA-treated BM-MSCs compared with BM-MSCs (Fig. 3B). Further, the protein level of p53 and p21 was greatly upregulated compared with BM-MSCs (Fig. 3B). We then performed SA-β-gal staining to detect senescent MSCs among the two groups. Ndufs6-siRNA-treated BM-MSCs exhibited increased β-gal positivity compared with BM-MSCs (Fig. 3C, D). Moreover, Ki-67 staining demonstrated that the number of Ki-67-positive cells was significantly reduced in Ndufs6-siRNA-treated BM-MSCs (Fig. 3E, F). Overall, these findings showed that knockdown of Ndufs6 enhanced cellular senescence of BM-MSCs.

### Replenishment of Ndufs6 in Ndufs6^−/−^-BM-MSCs largely reverses cellular senescence

**Fig. 4:**
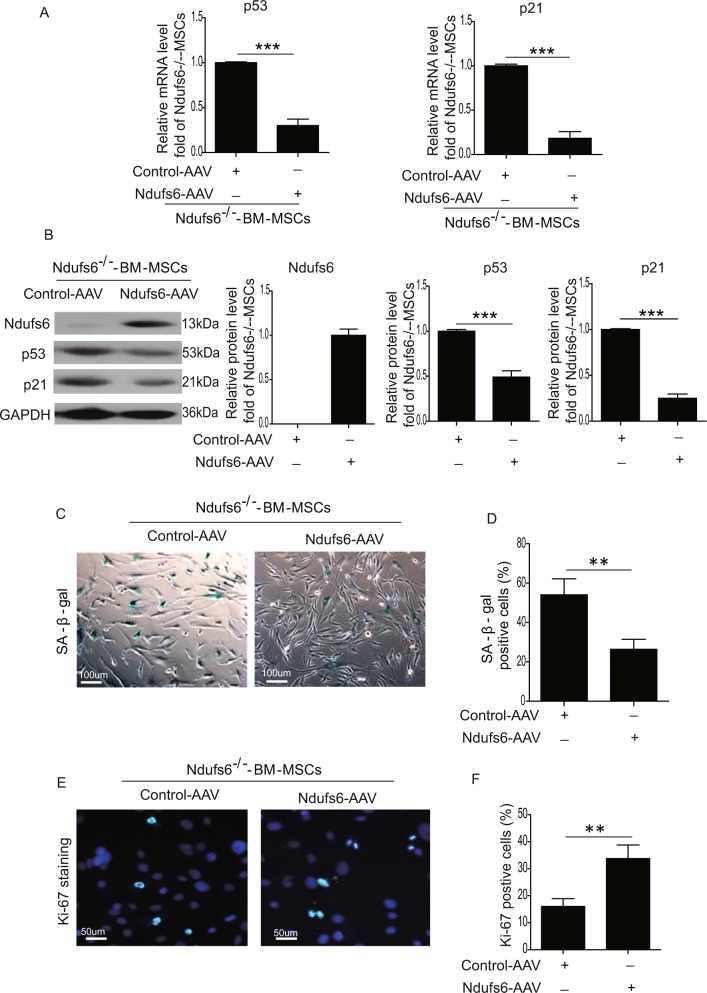
Overexpression of Ndufs6 in Ndufs6^−/−^-BM-MSCs decreases cellular senescence. **A** The mRNA level of Ndufs6, p53, and p21 was detected by RT-PCR in Ndufs6^−/−^-BM-MSCs and Ndufs6-AAV2-treated Ndufs6^−/−^-BM-MSCs. **B** The protein level of Ndufs6, p53, and p21 was detected by western blotting in Ndufs6^−/−^-BM-MSCs and Ndufs6-AAV-treated Ndufs6^−/−^BM-MSCs. **C** SA-β-gal staining showing β-gal-positive cells in Ndufs6^−/−^-BM-MSCs and Ndufs6-AAV-treated Ndufs6^−/−^BM-MSCs. **D** The β-gal positivity in Ndufs6^−/−^-BM-MSCs and Ndufs6-AAV-treated Ndufs6^−/−^BM-MSCs was quantified by calculating the ratio of stained cells to total cells. **E** The proliferation of Ndufs6^−/−^-BM-MSCs and Ndufs6-AAV-treated Ndufs6^−/−^BM-MSCs was assessed by Ki-67 staining. **F** The Ki-67 positivity in Ndufs6^−/−^-BM-MSCs and Ndufs6-AAV-treated Ndufs6^−/−^BM-MSCs was quantified by calculating the ratio of stained cells to total cells. Results are presented as mean ± SD. *n* = 3. **p < 0.01; ***p < 0.001.

To restore Ndufs6 in Ndufs6^−/−^-BM-MSCs, Ndufs6^−/−^-BM-MSCs were transduced with AAV containing Ndufs6. As expected, the mRNA level of p53 and p21 was much lower in Ndufs6-AAV-treated Ndufs6^−/−^BM-MSCs than control-AAV-treated Ndufs6^−/−^-BM-MSCs (Fig. 4A). Western blotting showed that the protein level of Ndufs6 was notably enhanced, whereas the protein level of p53 and p21 was significantly downregulated in Ndufs6-AAV-treated Ndufs6^−/−^-BM-MSCs compared with BM-MSCs (Fig. 4B). Next, we performed SA-β-gal staining to examine cellular senescence. As shown in Fig. 4C, D, the number of SA-β-gal-positive cells was dramatically reduced in Ndufs6^−/−^-BM-MSCs with Ndufs6 replenishment compared with Ndufs6^−/−^-BM-MSCs (Fig. 4C, D). The results were further confirmed by Ki-67 staining that revealed a significant increase of Ki-67 in Ndufs6^−/−^-BM-MSCs with Ndufs6 overexpression (Fig. 4E, F). Furthermore, we also examined whether restoring Ndufs6 expression could revert the reduced differentiation capacity of Ndufs6^−/−^-BM-MSCs. The differentiation capacity of Ndufs6^−/−^-BM-MSCs and Ndufs6^−/−^-BM-MSCs with Ndufs6 overexpression for adipogenesis, chondrogenesis, and osteogenesis was evaluated by measuring the mRNA level of the genes related to adipogenesis (LPL and Pparg), chondrogenesis (Acan and Col2a1), and osteogenesis (Bglap and Alpl). As shown in Fig. S5, the mRNA expression of LPL, Pparg, Acan, Col2a1, Bglap, and Alpl was greatly increased in Ndufs6^−/−^-BM-MSCs with Ndufs6 overexpression in differentiation conditions compared with that of Ndufs6^−/−^-BM-MSCs, suggesting that restoring Ndufs6 expression could revert the reduced differentiation capacity of Ndufs6^−/−^-BM-MSCs (Figure S5A–C). Taken together, these findings suggest that restoration of Ndufs6 in Ndufs6^−/−^-BM-MSCs reduced cellular senescence.

### Loss of Ndufs6 results in complex I deficiency and enhanced ROS generation

**Fig. 5:**
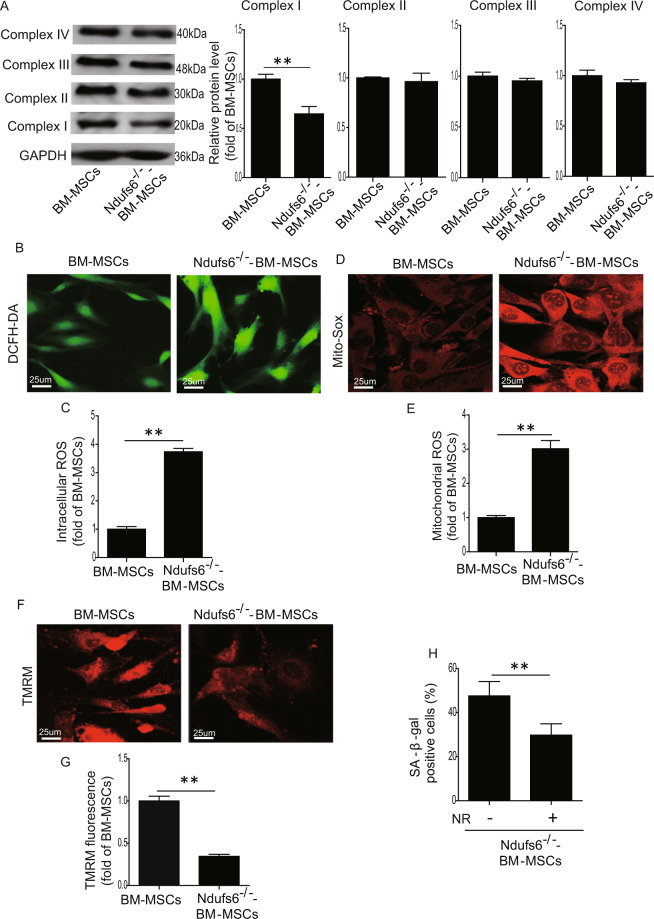
Ndufs6^−/−^-BM-MSCs exhibit complex I deficiency and enhanced ROS generation. **A** The protein level of complexes I, II, III, and IV was evaluated in BM-MSCs and Ndufs6^−/−^-BM-MSCs. **B** The intracellular ROS of BM-MSCs and Ndufs6^−/−^-BM-MSCs was measured by DCFH-DA staining. **C** The intracellular ROS of BM-MSCs and Ndufs6^−/−^-BM-MSCs was statistically quantified. **D** The mitochondrial ROS of BM-MSCs and Ndufs6^−/−^-BM-MSCs was measured by Mito-Sox staining. **E** The mitochondrial ROS of BM-MSCs and Ndufs6^−/−^-BM-MSCs was statistically quantified. **F** The MMP of BM-MSCs and Ndufs6^−/−^-BM-MSCs was assessed by TMRM staining. **G** The MMP of BM-MSCs and Ndufs6^−/−^-BM-MSCs was statistically quantified. **H** NR treatment decreased cellular senescence in Ndufs6^−/−^-BM-MSCs. Results are presented as mean ± SD. *n* = 3. **p < 0.01.

Since Ndufs6 is a major assembly subunit of complex I, absence of Ndufs6 may impair the activity of complex I. Western blotting results showed a reduced amount of complex I in Ndufs6^−/−^-BM-MSCs compared with BM-MSCs, presumably due to the absence of Ndufs6 (Fig. 5A). Nonetheless the protein level of complexes II, III, and IV did not differ significantly between the two (Fig. 5A), suggesting that knockout of Ndufs6 affects the function of complex I. Since complex I is closely linked to ROS generation, we subsequently examined ROS generation in Ndufs6^−/−^-BM-MSCs. DCFH-DA staining showed that Ndufs6^−/−^-BM-MSCs had an increased level of intracellular ROS compared with BM-MSCs (Fig. 5B, C). Flow cytometry analysis also showed a higher level of intracellular ROS in Ndufs6^−/−^-BM-MSCs than BM-MSCs (Fig. S6A). Moreover, Mito-Sox staining demonstrated that the level of mitochondrial ROS was greatly enhanced in Ndufs6^−/−^-BM-MSCs compared with BM-MSCs (Fig. 5D, E). Similarly, flow cytometry analysis also confirmed that the level of mitochondrial ROS was much higher in Ndufs6^−/−^-BM-MSCs than BM-MSCs (Fig. S6B). Next, TMRM staining was employed to detect the MMP in MSCs. It showed a significantly reduced TMRM fluorescence in Ndufs6^−/−^-BM-MSCs compared with BM-MSCs (Fig. 5F, G), indicating loss of MMP in Ndufs6^−/−^-BM-MSCs. Consistently, flow cytometry analysis also confirmed that the level of MMP was much lower in Ndufs6^−/−^-BM-MSCs than BM-MSCs (Fig. S6C). Overall, these results demonstrated that an absence of Ndufs6 enhances ROS generation and damages mitochondria via complex I deficiency. Since complex I mediates mitochondrial function, we thus determined whether absence of Ndufs6 can influence mitochondrial morphology and autophagy in Ndufs6^−/−^-BM-MSCs, using a TEM. As shown in Fig. S7, compared with BM-MSCs, the mitochondrial length was greatly increased in Ndufs6^−/−^-BM-MSCs (Fig. S7A, B). However, the number of autophagosomes in Ndufs6^−/−^-BM-MSCs was much lower than that in BM-MSCs (Fig. S7A, C). Given that complex I deficiency could result in NADH accumulation, whether NAD supplementation could rescue Ndufs6^−/−^-BM-MSC senescence has not been determined. Therefore, we treated the Ndufs6^−/−^-BM-MSCs with 20 mM nicotinamide riboside (NR) for 48 h and then assessed the cellular senescence. As shown in Fig. 5H, compared with Ndufs6^−/−^-BM-MSCs, the cellular senescence was greatly reduced in NR-treated Ndufs6^−/−^-BM-MSCs, indicating NR treatment could ameliorate senescence of Ndufs6-deficient MSCs (Fig. 5H).

### Downregulation of Ndufs6 induces cell senescence of MSCs via regulation of the ROS/p53 pathway

**Fig. 6:**
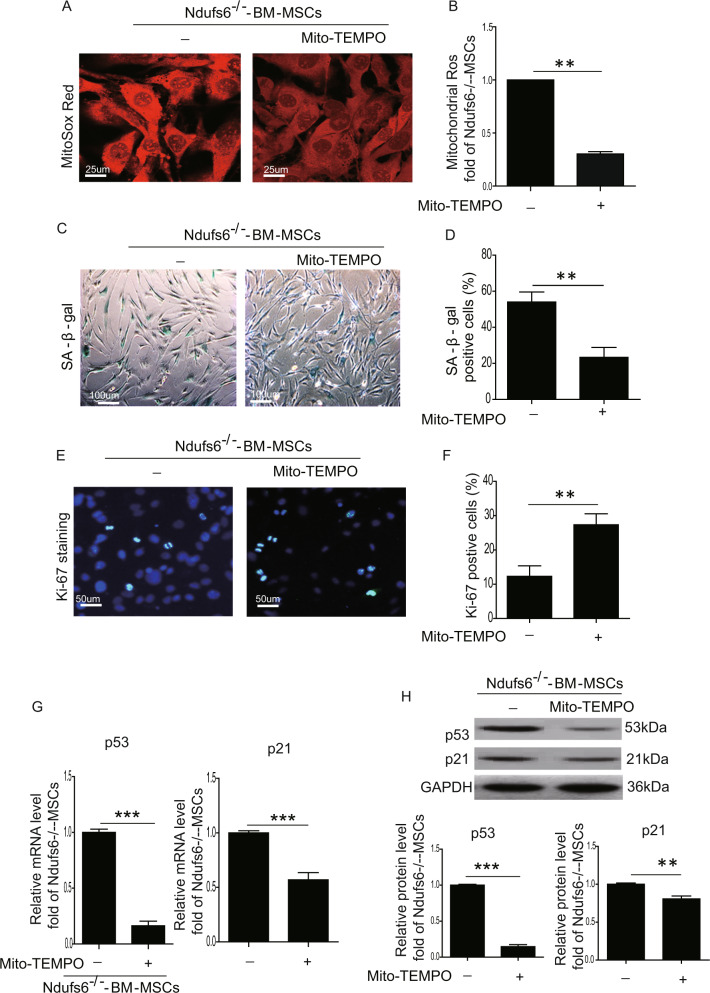
Inhibition of ROS decreases cellular senescence of Ndufs6^−/−^-BM-MSCs. **A** The mitochondrial ROS of Ndufs6^−/−^-BM-MSCs and Mito-TEMPO-treated Ndufs6^−/−^-BM-MSCs was measured by Mito-Sox staining. **B** The mitochondrial ROS of Ndufs6^−/−^-BM-MSCs and Mito-TEMPO-treated Ndufs6^−/−^-BM-MSCs was statistically quantified. **C** SA-β-gal staining showing β-gal-positive cells in Ndufs6^−/−^-BM-MSCs and Mito-TEMPO-treated Ndufs6^−/−^-BM-MSCs. **D** The β-gal positivity in Ndufs6^−/−^-BM-MSCs and Mito-TEMPO-treated Ndufs6^−/−^-BM-MSCs was quantified by calculating the ratio of stained cells to total cells. **E** The proliferation of Ndufs6^−/−^-BM-MSCs and Mito-TEMPO-treated Ndufs6^−/−^-BM-MSCs was assessed by Ki-67 staining. **F** The Ki-67 positivity in Ndufs6^−/−^-BM-MSCs and Mito-TEMPO-treated Ndufs6^−/−^-BM-MSCs was quantified by calculating the ratio of stained cells to total cells. **G** The mRNA level of p53 and p21 was assessed in Ndufs6^−/−^-BM-MSCs and Mito-TEMPO-treated Ndufs6^−/−^-BM-MSCs. **H** The protein level of p53 and p21 was assessed in Ndufs6^−/−^-BM-MSCs and Mito-TEMPO-treated Ndufs6^−/−^-BM-MSCs. Results are presented as mean ± SD. *n* = 3. **p < 0.01.

To verify whether the absence of Ndufs6 induces cell senescence of MSCs via regulation of ROS, we used the mitochondrial ROS inhibitor Mito-TEMPO (Santa Cruz, SC-221945, 100 μM) to treat Ndufs6^−/−^-BM-MSCs for 48 hours. We first examined the cellular proliferation of Ndufs6^−/−^-BM-MSCs and Mito-TEMPO–treated Ndufs6^−/−^-BM-MSCs, and confirmed that the cellular proliferation of Mito-TEMPO-treated Ndufs6^−/−^-BM-MSCs was significantly increased (Fig. S8). Mito-TEMPO-treated Ndufs6^−/−^-BM-MSCs exhibited a decreased level of mitochondrial ROS compared with Ndufs6^−/−^-BM-MSCs (Fig. 6A, B). SA-β-gal staining showed that the SA-β-gal activity was dramatically reduced in Mito-TEMPO-treated Ndufs6^−/−^-BM-MSCs compared with Ndufs6^−/−^-BM-MSCs (Fig. 6C, D). Furthermore, the number of Ki-67-positive cells was significantly enhanced in Mito-TEMPO-treated Ndufs6^−/−^-BM-MSCs compared with Ndufs6^−/−^-BM-MSCs (Fig. 6E, F). Subsequently, we compared the mRNA level of p53 and p21 in Mito-TEMPO-treated Ndufs6^−/−^-BM-MSCs and Ndufs6^−/−^-BM-MSCs, and found that the mRNA of p53 and p21 was much lower in Mito-TEMPO-treated Ndufs6^−/−^-BM-MSCs (Fig. 6G). Consistently, the protein level of p53 and p21 was also greatly downregulated in Mito-TEMPO-treated Ndufs6^−/−^-BM-MSCs compared with BM-MSCs (Fig. 6h). To further evaluate the effects of Mito-TEMPO, we detected oxygen consumption rate (OCR) of BM-MSCs, Ndufs6^−/−^-BM-MSCs, and Mito-TEMPO-treated Ndufs6^−/−^-BM-MSCs, using an extracellular flux analyzer (Fig. S9A). Compared with BM-MSCs, basal mitochondrial OCR and ATP production significantly reduced in Ndufs6^−/−^-BM-MSCs (Fig. S9B, C). However, basal mitochondrial OCR and ATP production were markedly improved in Ndufs6^−/−^-BM-MSCs with Mito-TEMPO treatment compared with Ndufs6^−/−^-BM-MSCs (Fig. S9B, C). These results demonstrate that inhibition of mitochondrial ROS alleviated cell senescence of Ndufs6^−/−^-BM-MSCs, indicating that cellular senescence in MSCs due to Ndufs6 is associated with the ROS/p53/p21 signaling pathway.

## Discussion

There are several major findings of this study. First, we have determined that a mitochondrial complex I accessory subunit, Ndufs6, is notably downregulated in both human and mice-aged MSCs. Second, we have verified that downregulation of Ndufs6 is not only a consequence of MSC ageing, but also accelerates MSC ageing, thus downregulation of Ndufs6 and MSC ageing creates a vicious circle. Third, we established that depressed Ndufs6 leads to complex I functional deficiency, and activates excessive ROS and p53/p21, thus accelerating cellular senescence of MSCs. Fourth, inhibition of ROS or replenishment of Ndufs6 attenuates absence of Ndufs6-induced cellular senescence.

Over the past decades, the senescence of MSCs has attracted huge attention due to their critical role in regenerative medicine. Senescent MSCs exhibit decrease differentiation capacity, anti-inflammation capacity, and reduced immunomodulatory activity^30,32–34^. Understanding the potential mechanisms that underlie MSC senescence is essential to the development of novel strategies to improve MSC quality, especially those from aged donors. It has been well documented that genetic alterations to MSCs contributes to cellular senescence. UBC knockdown induces senescence of MSCs and impairs their functions, whereas UBC overexpression ameliorates MSC senescence and improves their proliferative activity^35^. Conditional knockdown of Foxp1 in MSCs leads to premature senescence as evidenced by impaired bone mass and reduced MSC self-renewal capacity^36^. Knockout of WT p53-inducible phosphatase-1 in BM-MSCs results in premature characteristics of senescence, including a typical senescent morphology, increased β-gal activity, and decreased proliferative capacity^37^. Given the known evidence that a genetic defect is closely linked to cellular senescence, we sought to determine whether Ndufs6 mediates senescence in MSCs. We found that the differentiation capacity was greatly reduced in Ndufs6^−/−^-BM-MSCs compared with WT MSCs. Moreover, Ndufs6^−/−^-BM-MSCs exhibited enhanced β-gal activity and reduced proliferative ability, suggesting that Ndufs6 may be involved in cellular senescence. Nonetheless it has yet to be shown how Ndufs6 mediates MSC senescence.

Complex I is the major carrier of electron and subsequent superoxide production in mitochondrial membrane^38^. Complex I is the most vulnerable enzyme and the first to be damaged in many mitochondrial disorders. It has also been suggested that complex I malfunction is involved in the pathogenesis of diabetes and ageing. Nonetheless it is composed of at least 45 different subunits whose primary structures have only just been determined^39^. Understanding how the subunits of complex I contribute to stem cell ageing is just the beginning. Many subunits of complex I have been referred to as accessory (or supernumerary) subunits. Initially, these accessory subunits have been considered to be nonessential to the structure and function of mitochondrial complex I. Nonetheless recent research has questioned whether these “accessory subunits” are really accessory. Some of these subunits including Ndufs6 are absolutely essential for complex I function and loss or mutations of these subunits can lead to complex I defects. Mutations of Ndufa9, a Q-module subunit for complex I, cause mitochondrial complex I assembly defect, leading to a severe and fatal neonatal phenotype^40^. Mutations in Ndufb10, an accessory subunit of complex I, results into complex I deficiency, thus impairing the oxidation^41^. Conditional ablation of Ndufa5 induces partial complex I deficiency, leading to lethargy and loss of motor skills in mice^42^. In the current study, following bioinformatics analysis that Ndufs6 downregulation is a hallmark of BM-MSCs ageing, we verified that Ndufs6 is also an inducer of MSC ageing, closely associated with downregulated Ndufs6-provoked complex I deficiency.

Accumulating evidence has demonstrated that complex I deficiency can cause increased ROS generation, leading to several devastating mitochondrial disorders. Leman et al. analyzed several fibroblast cell lines isolated from patients with inherited complex I deficiency and found that ROS was greatly enhanced^43^. Knockout of Ndufs4-induced complex I deficiency triggers ROS generation and lipid droplet accumulation in glia, and subsequent neurodegenerative disease in mice^44^. Accordingly, we also observed elevated ROS in Ndufs6^−/−^-BM-MSCs compared with BM-MSCs. Further, Ndufs6-siRNA-treated BM-MSCs exhibited complex I deficiency and enhanced ROS, suggesting that complex I deficiency contributes to ROS generation. It is well established that ROS can trigger cellular senescence via regulation of multiple pathways. Inflammatory cytokine TNF-α can activate the ROS/NF-κB pathway to induce nucleus pulposus cell senescence^45^. Iron overload-induced ROS accumulation induces BM-MSC senescence via upregulation of the protein expression of p53, ERK, and p38 (ref. ^46^). Simvastatin treatment can induce senescence of human melanoma cells via activation of the ROS/p53/p21 signaling pathway^47^. In this study, we used Mito-TEMPO to treat Ndufs6^−/−^-BM-MSCs, and found that cellular senescence was greatly reduced along with a decreased level of ROS and protein level of p53/p21, indicating that the ROS/p53/p21 signaling pathway is involved in Ndufs6 knockout-induced cellular senescence of MSCs.

In summary, based on the above findings, we conclude that depressed Ndufs6 and MSC ageing interact in a manner of reciprocal causation. Ndufs6 defect impairs complex I function and activates the ROS/p53/p21 signaling pathway, leading to cellular senescence of MSCs. Our study provides novel insight to precisely target the mitochondria subunit to prevent senescence of MSCs.

## Supplementary information

Supplemental Figure legends

Figure s1

Figure S2

Figure S3

Figure S4

Figure S5

Figure S6

Figure S7

Figure S8

Figure S9
