# Supplementary material for: Adult mesenchymal stem cell ageing interplays with depressed mitochondrial Ndufs6
Source: Cell Death Dis. 2020 Dec 15;11(12):1075. doi: 10.1038/s41419-020-03289-w (PMC7738680; doi:10.1038/s41419-020-03289-w)
Supplement: Supplementary file 1 — Supplemental Figure legends [file 41419_2020_3289_MOESM1_ESM.docx]

**Supplemental figure legends:**

**Figure S1. The plasmid map of AAV2-Ndufs6.**

**Figure S2: mRNA expression change of NDUFS1-8 genes during aging in human or mouse.** Five GEO microarray datasets were used. (A) GSE9593 representing early and senescent MSC passages. (B) GSE35959 representing human MSCs from young, aging and primary osteoporosis. (C) GSE70376 representing muscle stem cells in homeostatic conditions or after cardiotoxin injury. (D) GSE47177 representing quiescent satellite cells from hindlimb muscle of uninjured young or old mice. (E-F) GSE56560 representing young and senescent human MSC for two donors, respectively.

**Figure S3. The mRNA level of Ndufs6 in aged-BM-MSCs and young-BM-MSCs derived from mice.** RT-PCR results showed that the mRNA level of Ndufs6 was much lower in aged-BM-MSCs derived from mice than young-BM-MSCs. Results are presented as mean±SD. n=3. ****p<0.001*.

**Figure S4. Characterizations of BM-MSCs and Ndufs6^-/-^-BM-MSCs**

(A) Representative FASC showing the positive expression of surface markers SCA1, CD90, and CD105 and negative expression of CD34, CD45 in BM-MSCs and Ndufs6^-/-^-BM-MSCs at passage 4. (B) The protein level of Ndufs6 in BM-MSCs and Ndufs6^-/-^-BM-MSCs was determined by western blotting. (C) The differentiation capacity of adipogenesis, chondrogenesis and osteogenesis of BM-MSCs and Ndufs6^-/-^-BM-MSCs was confirmed by Oil Red staining, Alcian Blue staining and Alizarin Red staining, respectively. (D, E, F) The mRNA expressions of LPL, Pparg, Acan, Col2a1, Bglap and Alpl were detected in BM-MSCs and Ndufs6^-/-^-BM-MSCs after differentiation. Results are presented as means±SD. n=3. ***p*<0.01; ****p*<0.001.

**Figure S5. Evaluation of the differentiation capacity of Ndufs6^-/-^-BM-MSCs and Ndufs6^-/-^-BM-MSCs with Ndufs6 overexpression.**

(A, B, C) The differentiation capacity of Ndufs6^-/-^-BM-MSCs and Ndufs6^-/-^-BM-MSCs with Ndufs6 overexpression (Ndufs6-Ndufs6^-/-^-BM-MSCs) for adipogenesis, chondrogenesis and osteogenesis was evaluated by measuring the mRNA level of the genes related to adipogenesis (LPL, Pparg), chondrogenesis (Acan, Col2a1) and osteogenesis (Bglap, Alpl). Results are presented as means±SD. n=3. ***p*<0.01

**Figure S6. Flow cytometry analysis of intracellular ROS, mitochondrial ROS and MMP in BM-MSCs and Ndufs6^-/-^-BM-MSCs.**

(A) Intracellular ROS level was measured with DCFH-DA staining by flow cytometry analysis in BM-MSCs and Ndufs6^-/-^-BM-MSCs. BM-MSCs were treated with H_2_O_2_ for ROS generation as positive control. (B) Mitochondrial ROS level was measured with MitoSox staining by flow cytometry analysis in BM-MSCs and Ndufs6^-/-^-BM-MSCs. BM-MSCs treated with antimycin A were used as positive control. (C) MMP level was measured with TMRM staining by flow cytometry analysis in BM-MSCs and Ndufs6^-/-^-BM-MSCs. BM-MSCs were treated with FCCP as positive control.

**Figure S7. TEM analysis of mitochondrial length and autophagy level in BM-MSCs and Ndufs6^-/-^-BM-MSCs.** (A) Representative images of mitochondrial length and autophagosomes in BM-MSCs and Ndufs6^-/-^-BM-MSCs examined by a TEM. Scale bar=1μM. Red arrows show mitochondria. Black arrows show autophagosomes. (B) Quantitative analysis of mitochondrial length in BM-MSCs and Ndufs6^-/-^-BM-MSCs. (C) Quantitative analysis of autophagosomes in BM-MSCs and Ndufs6^-/-^-BM-MSCs. Results are presented as means±SD. n=3. ****p*<0.001

**Figure S8. Mito-TEMPO treatment enhanced the cellular proliferation of Ndufs6^-/-^-BM-MSCs.** Results are presented as means±SD. n=3. ***p*<0.01

**Figure S9. The bioenergetic profile in BM-MSCs, Ndufs6^-/-^-BM-MSCs and Mito-TEMPO-treated Ndufs6^-/-^-BM-MSCs examined by an extracellular flux analyzer.** (A) OCR of BM-MSCs, Ndufs6^-/-^-BM-MSCs and Mito-TEMPO-treated Ndufs6^-/-^-BM-MSCs was measured over time (minutes). The injection order of Olig, FCCP, and Rot are shown. (B) Basal mitochondrial OCR in BM-MSCs, Ndufs6^-/-^-BM-MSCs and Mito-TEMPO-treated Ndufs6^-/-^-BM-MSCs was calculated. (C) ATP production in BM-MSCs, Ndufs6^-/-^-BM-MSCs and Mito-TEMPO-treated Ndufs6^-/-^-BM-MSCs was calculated. Results are presented as means±SD. n=3. ****p*<0.001.
