# Supplementary figures and images for: Adult mesenchymal stem cell ageing interplays with depressed mitochondrial Ndufs6
Source: Cell Death Dis. 2020 Dec 15;11(12):1075. doi: 10.1038/s41419-020-03289-w (PMC7738680; doi:10.1038/s41419-020-03289-w)

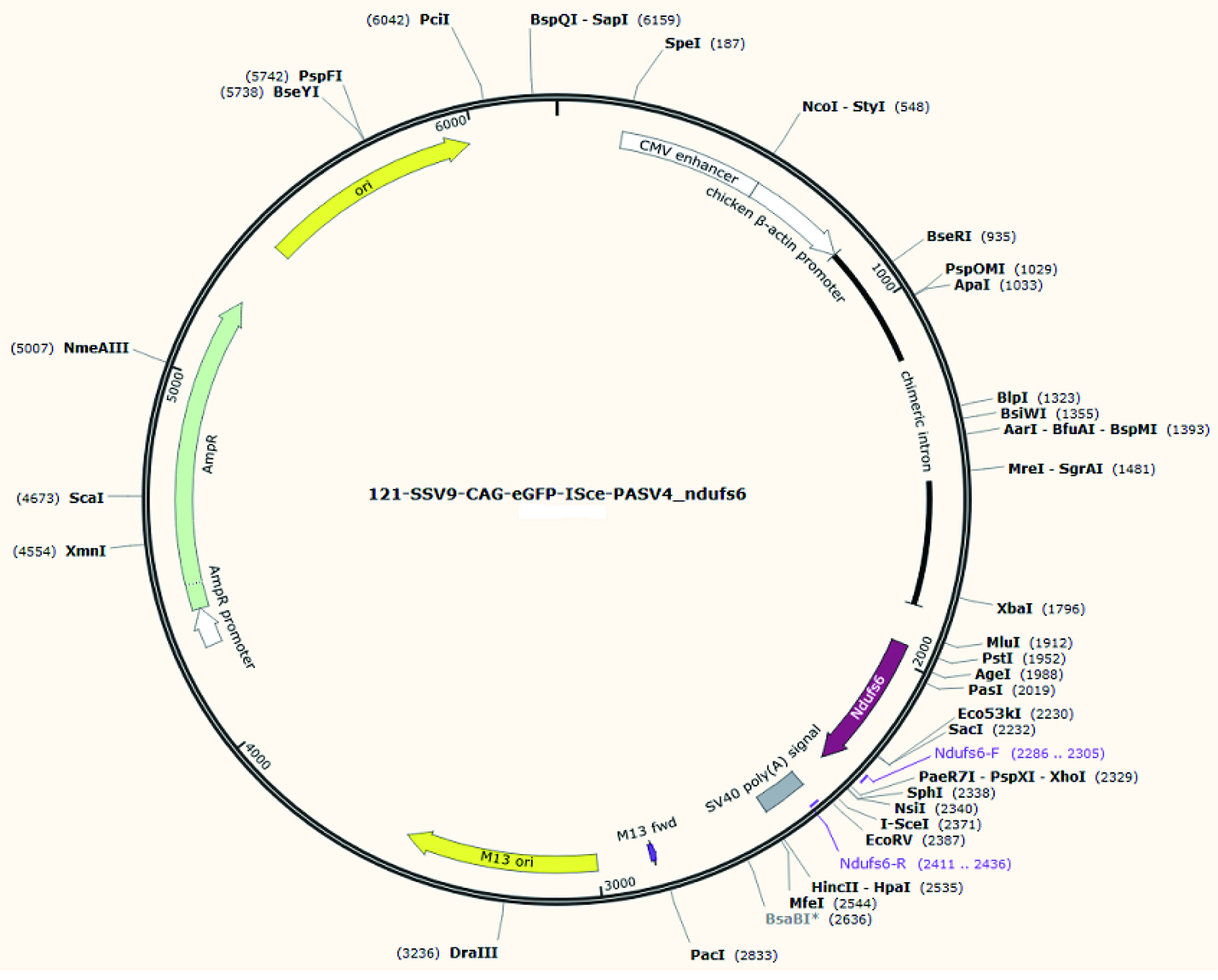

Supplement: Supplementary file 2 — Figure s1 [file 41419_2020_3289_MOESM2_ESM.tif]

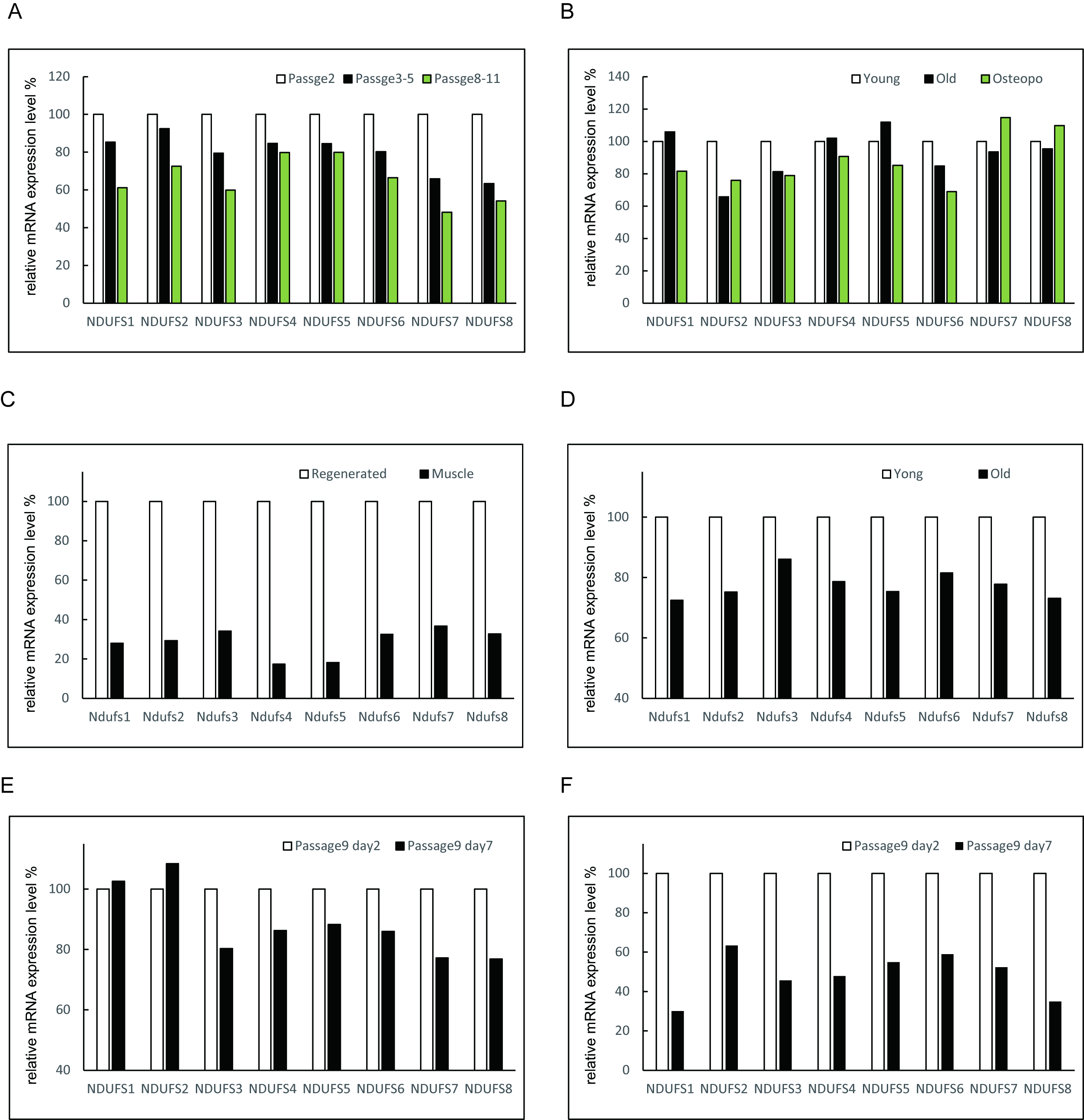

Supplement: Supplementary file 3 — Figure S2 [file 41419_2020_3289_MOESM3_ESM.tif]

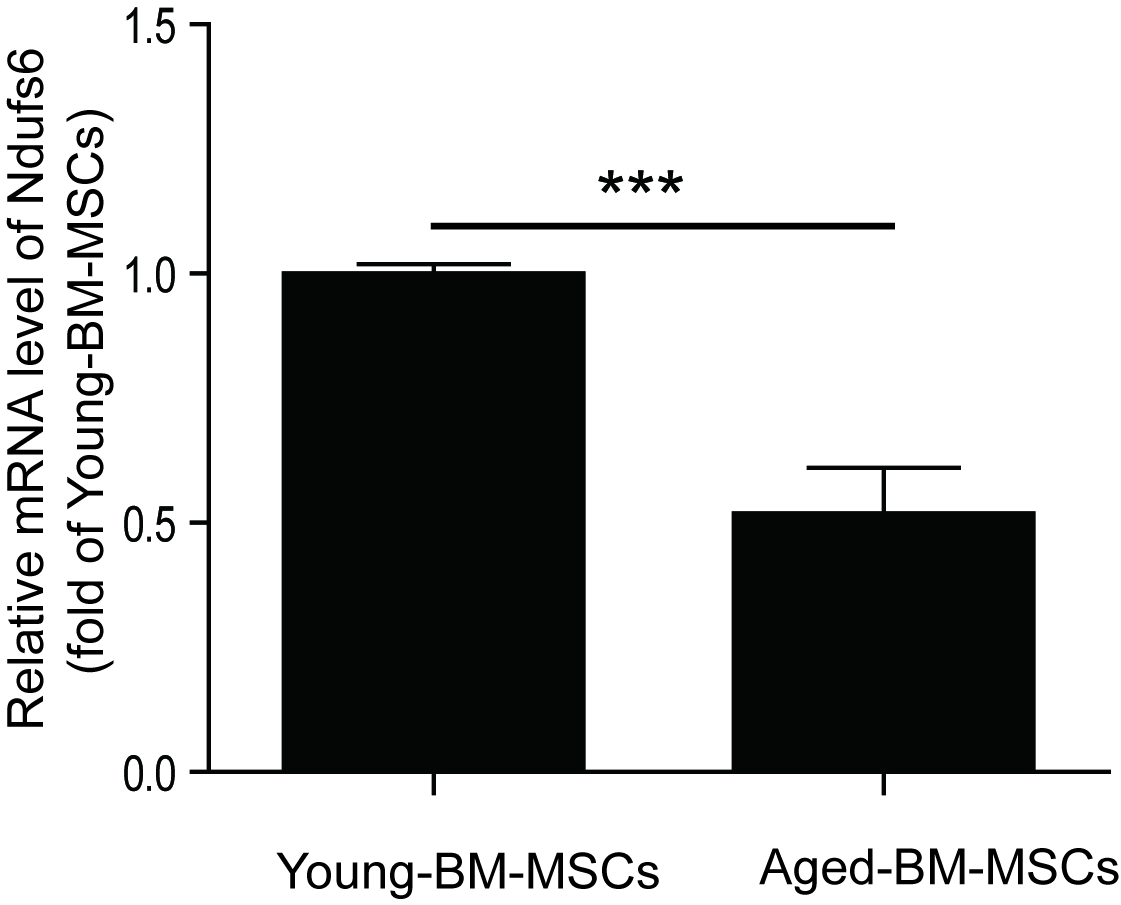

Supplement: Supplementary file 4 — Figure S3 [file 41419_2020_3289_MOESM4_ESM.tif]

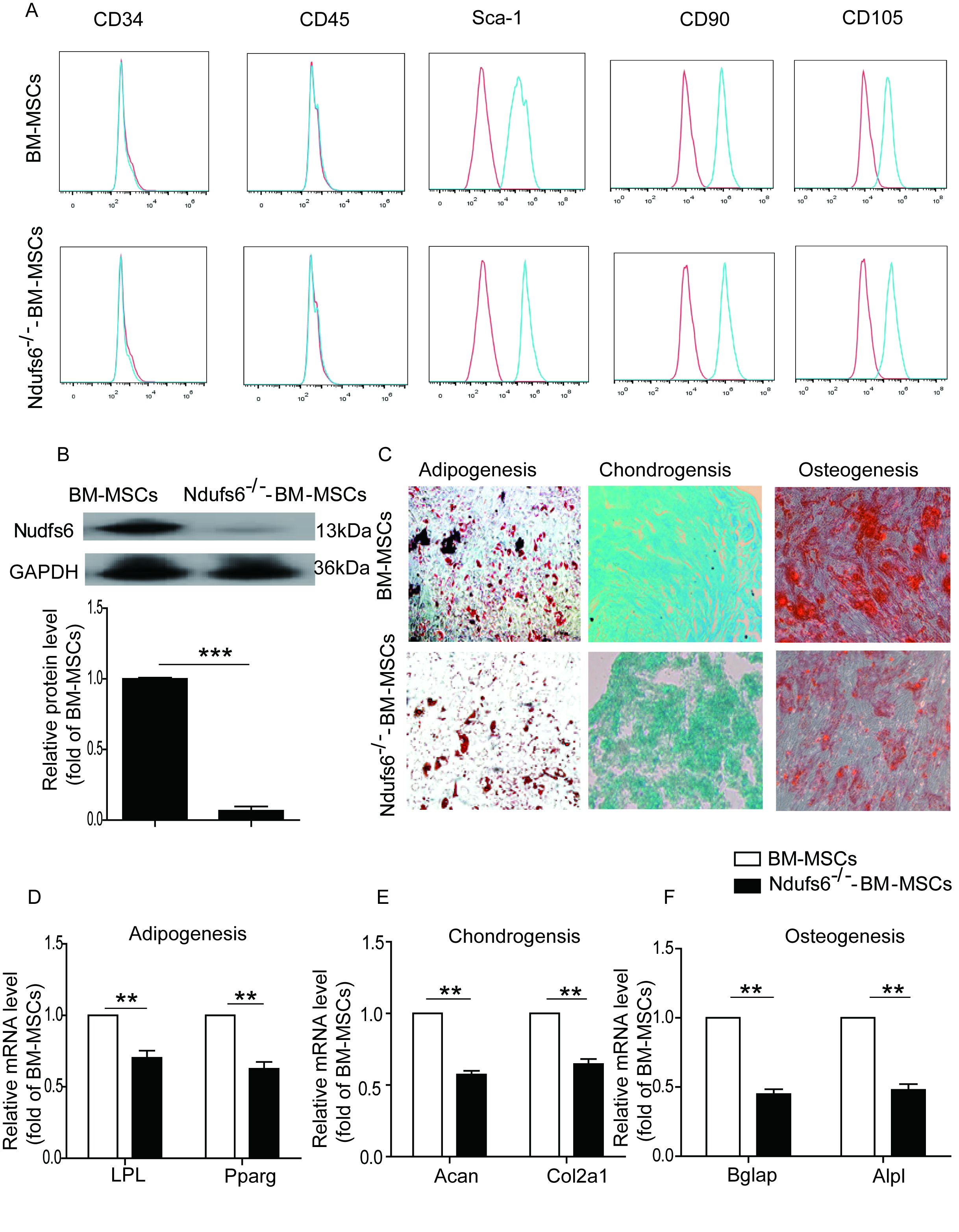

Supplement: Supplementary file 5 — Figure S4 [file 41419_2020_3289_MOESM5_ESM.tif]

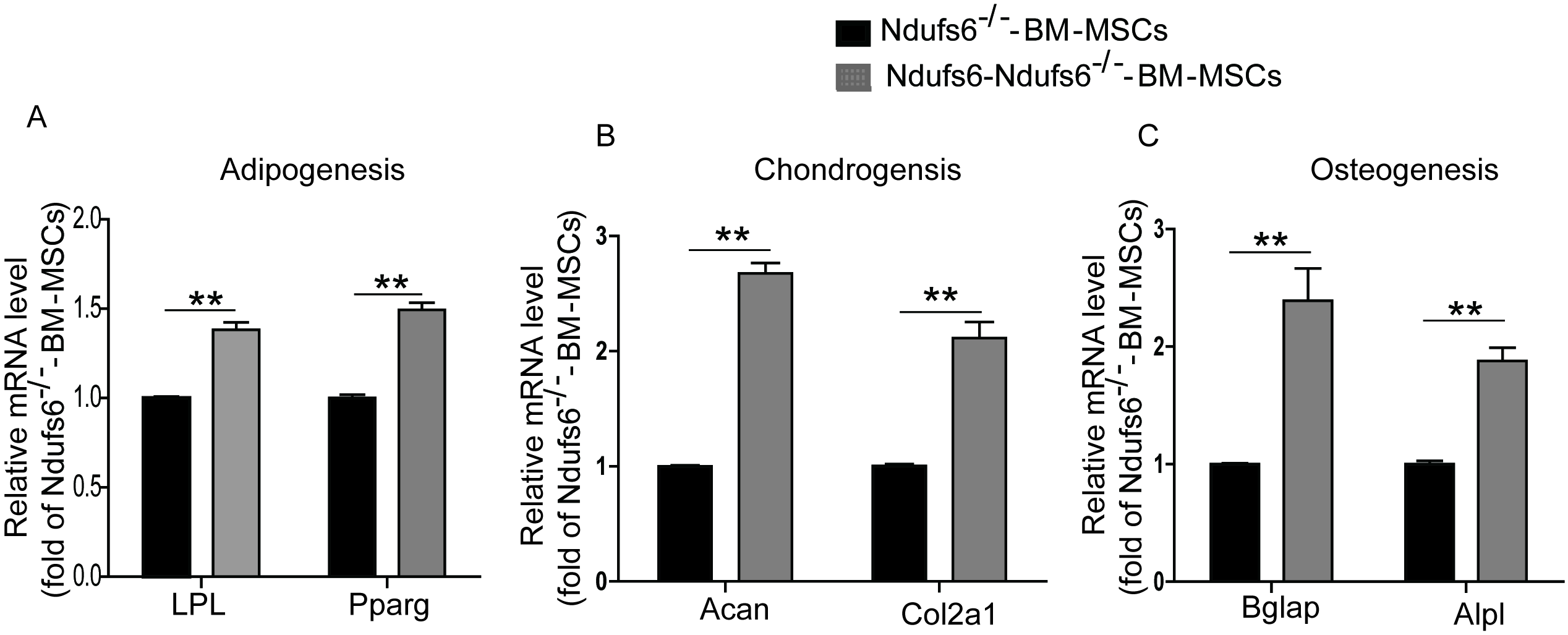

Supplement: Supplementary file 6 — Figure S5 [file 41419_2020_3289_MOESM6_ESM.tif]

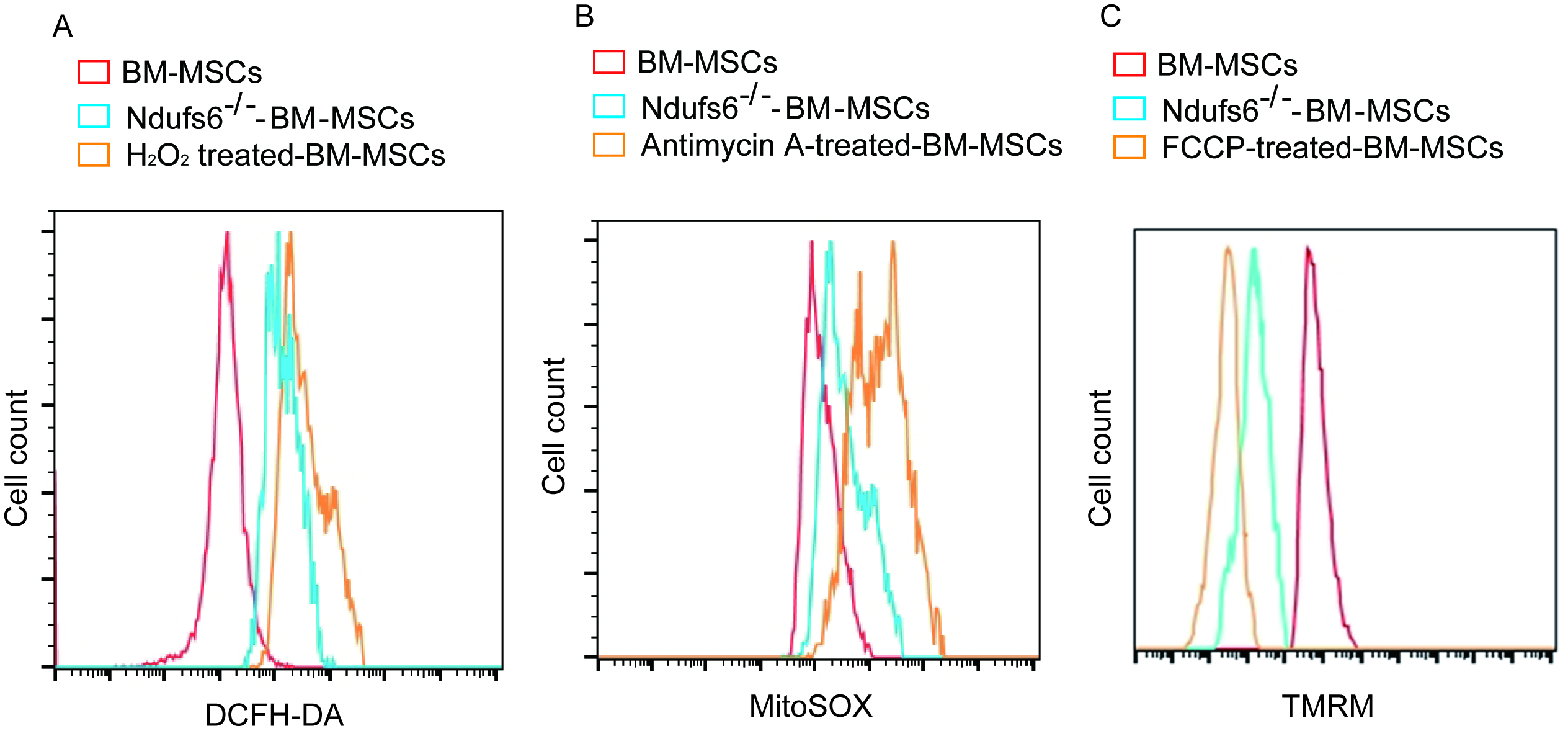

Supplement: Supplementary file 7 — Figure S6 [file 41419_2020_3289_MOESM7_ESM.tif]

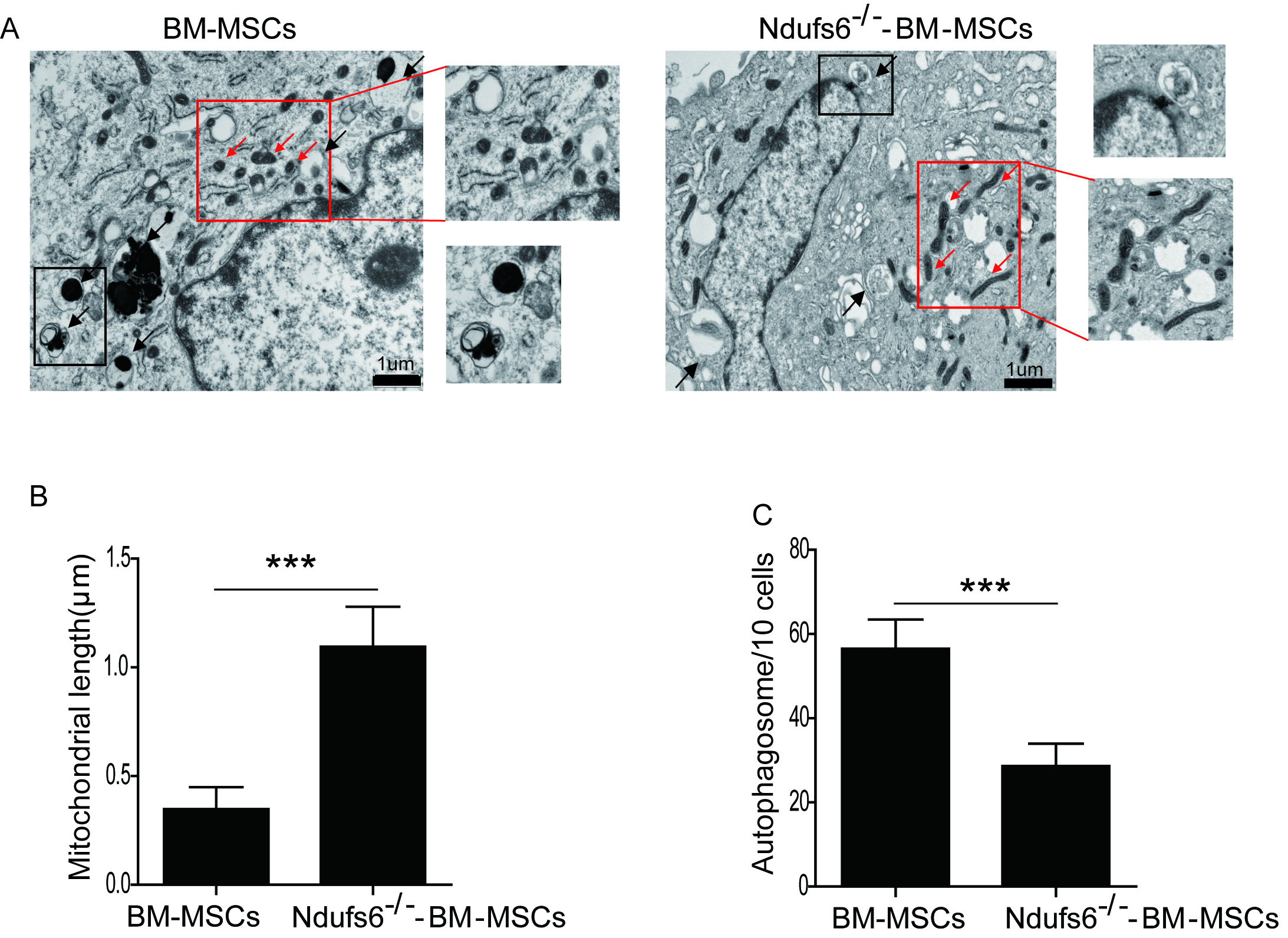

Supplement: Supplementary file 8 — Figure S7 [file 41419_2020_3289_MOESM8_ESM.tif]

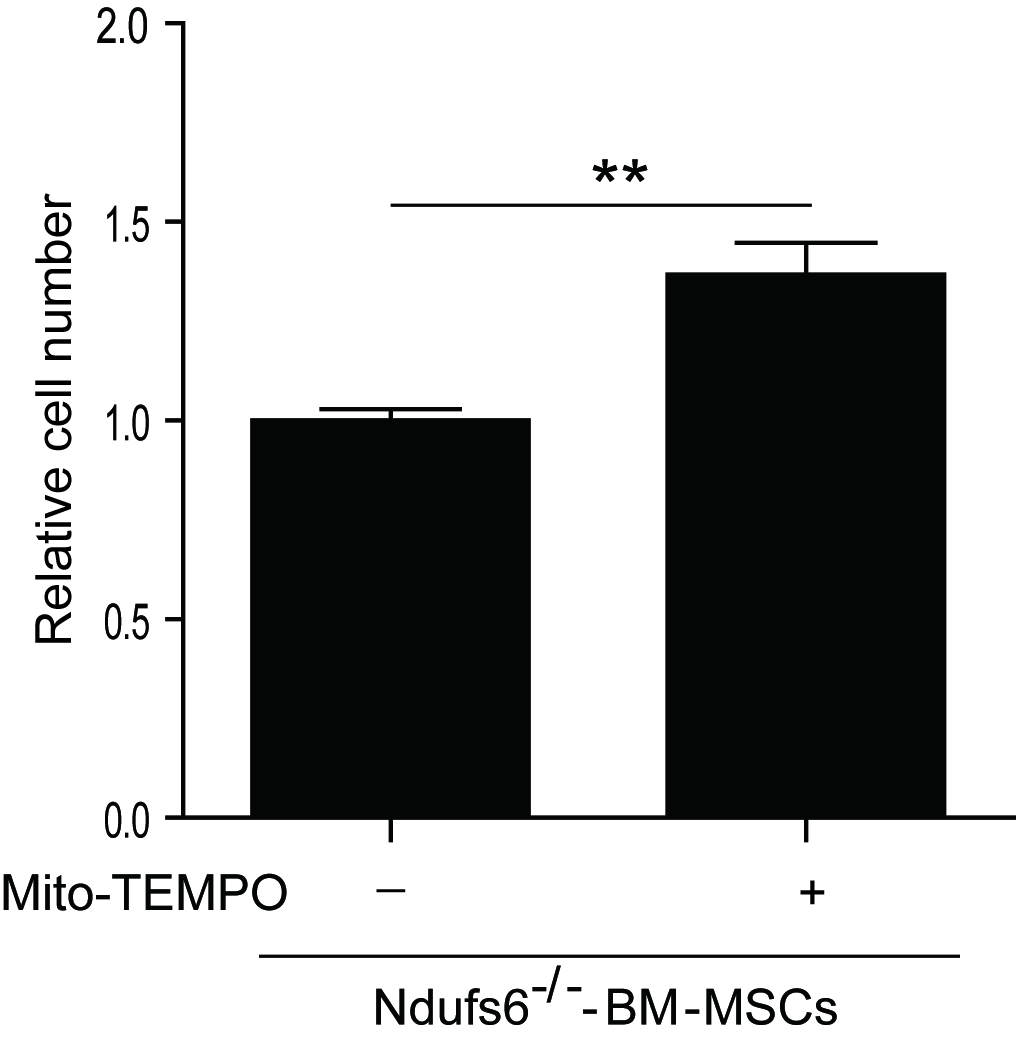

Supplement: Supplementary file 9 — Figure S8 [file 41419_2020_3289_MOESM9_ESM.tif]

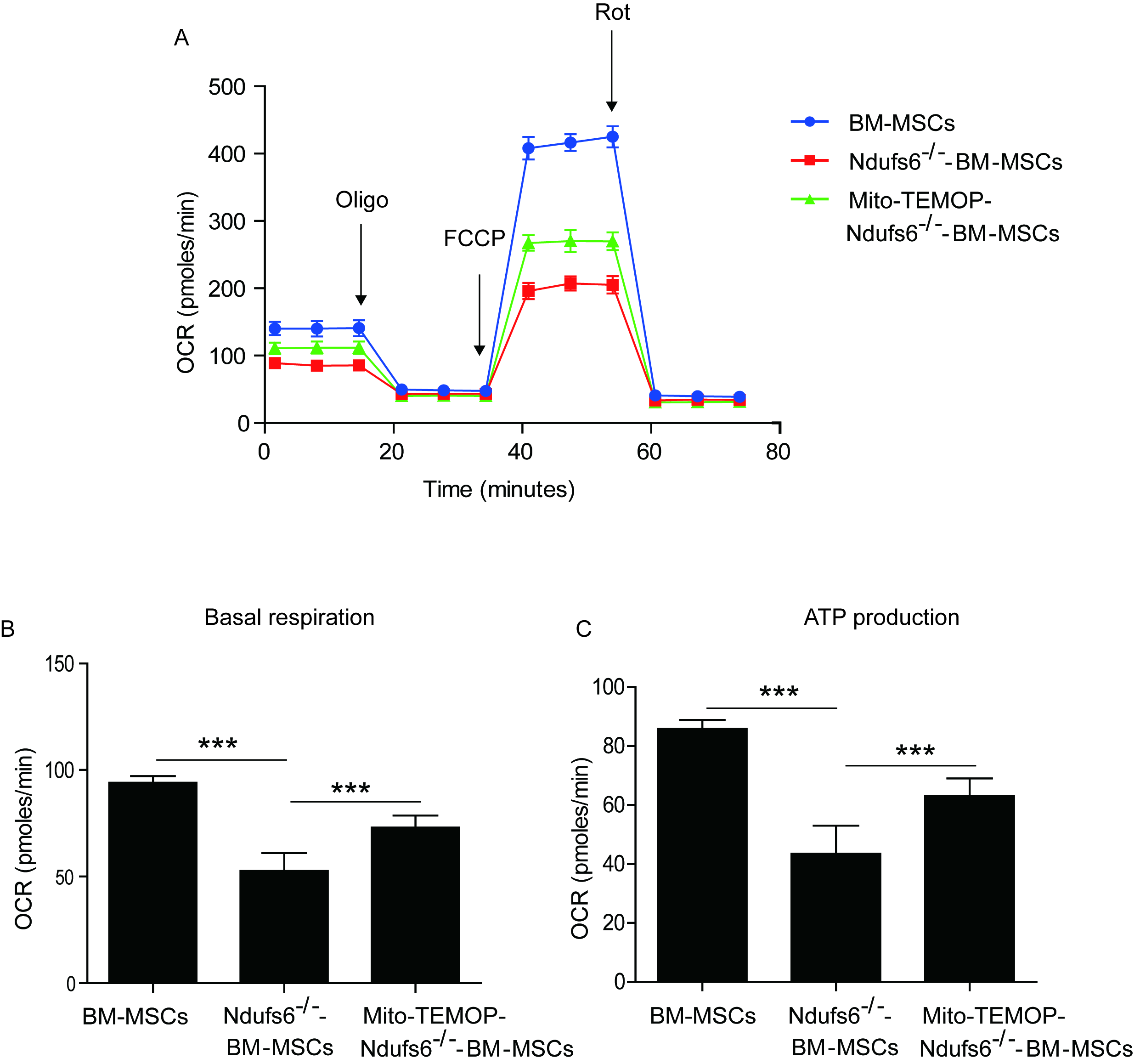

Supplement: Supplementary file 10 — Figure S9 [file 41419_2020_3289_MOESM10_ESM.tif]
